# HIV-1 replication changes the function of the PKR activator PACT

**DOI:** 10.1186/1742-4690-10-S1-P35

**Published:** 2013-09-19

**Authors:** Guerline Clerzius, Eileen Shaw, Aïcha Daher, Samantha Burugu, Jean-François Gélinas, Jean-Pierre Routy, Rekha C Patel, Anne Gatignol

**Affiliations:** 1 Virus-Cell Interactions Laboratory, Lady Davis Institute for Medical Research,, Montréal, QC, Canada; 2 Department of Medicine, Division of Experimental Medicine, McGill University, Montréal, QC, Canada; 3 Department of Microbiology and Immunology, McGill University, Montréal, QC, Canada; 4 Chronic Viral Illness Service and Division of Hematology, McGill University Health Centre, Montréal, QC, Canada; 5 Department of Biological Sciences, University of South Carolina, Columbia, SC, USA

## Background

HIV-1 translation is modulated by the activation of the interferon-inducible Protein Kinase R (PKR), which phosphorylates its downstream target, the eukaryotic translation Initiation Factor 2 (eIF2a). Phosphorylated eIF2a blocks translation initiation and consequently viral replication. PKR is not activated in HIV-1-replicating lymphocytes. Inactivated PKR allows high HIV-1 translation and may contribute to HIV-1 persistence in several cell types.

## Methods

Peripheral blood mononuclear cells (PBMCs) were infected with HIV-1 molecular clone pNL4-3. Viral kinetics were followed by reverse transcriptase (RT) assay. Expression of viral and cellular proteins were monitored by immunoprecipitation (IP) and western blots.

## Results

PKR is transiently induced and activated in PBMCs after HIV-1 infection and dephosphorylated during viral replication. The expression of two double-stranded RNA binding proteins, the RNA adenosine deaminase (ADAR) 1 and the PKR Activator (PACT) is induced during HIV-1 infection. By co-IP of HIV-1-infected lymphocytes with antibodies against PKR, we identified a multiprotein complex, which contains ADAR1 and PACT. PACT is known to activate PKR after a cellular stress. In cells transfected with an HIV-1 molecular clone, PACT unexpectedly inhibited PKR and eIF2a phosphorylation and increased HIV-1 protein expression and virion production. Short hairpin RNAs against PACT decreased HIV-1 protein expression. Furthermore, ADAR1, the TAR RNA binding protein, TRBP, and PACT all inhibit PKR and eIF2a phosphorylation in HIV-1-expressing cells. In the astrocytic cell line U251MG that weakly expresses TRBP, PACT also mediated an increased HIV-1 protein expression and a decreased PKR phosphorylation. Finally, PACT and ADAR1 interact with each other in the absence of RNA, which may mediate the change of PACT function in HIV-1 infected cells.

## Conclusions

In contrast to its previously described activity, PACT contributes to PKR dephosphorylation during HIV-1 replication. This activity is in addition to the previously described inhibition of PACT by TRBP [1] and to the direct activity of ADAR1 on PKR [2]. The change in PACT function is likely due to its interaction with ADAR1 but does not exclude the contribution of another HIV-1 component or virally-induced cellular factor. PKR inactivation likely contributes to HIV-1 persistence in several cell types.

## References

[B1] Daher A Laraki G Singh M Melendez-Peña CE Bannwarth S Peters AH Meurs EF Braun RE Patel RC Gatignol A TRBP control of PACT-induced phosphorylation of PKR is reversed by stress Mol Cell Biol 2009 29 254 265 10.1128/MCB.01030-08 18936160 PMC2612476

[B2] Clerzius G Gélinas JF Daher A Bonnet M Meurs EF Gatignol A ADAR1 interacts with PKR during human immunodeficiency virus infection of lymphocytes and contributes to viral replication J Virol 2009 83 10119 10128 10.1128/JVI.02457-08 19605474 PMC2747994

